# Molecular genetic detection and differentiation of Xanthomonas oryzae pv. oryzicola, bacterial leaf streak agents of rice

**DOI:** 10.18699/VJGB-22-66

**Published:** 2022-10

**Authors:** M.L. Koroleva, S.A. Blinova, A.A. Shvartsev, V.E. Kurochkin, Ya.I. Alekseev

**Affiliations:** Limited Liability Company “Syntol”, Moscow, Russia; Limited Liability Company “Syntol”, Moscow, Russia All-Russian Research Institute of Agricultural Biotechnology, Moscow, Russia; Limited Liability Company “Syntol”, Moscow, Russia; Institute for Analytical Instrumentation of the Russian Academy of Science, St. Petersburg, Russia; Limited Liability Company “Syntol”, Moscow, Russia Institute for Analytical Instrumentation of the Russian Academy of Science, St. Petersburg, Russia

**Keywords:** Xanthomonas oryzae pv. oryzicola, Xanthomonas, polymerase chain reaction, qPCR, bacterial leaf streak, sensitivity, species diagnostics, specif icity, Xanthomonas oryzae pv. oryzicola, Xanthomonas, полимеразная цепная реакция, ПЦР-РВ, бактериальная полосатость риса, специфичность, чувствительность, видовая диагностика

## Abstract

The genus Xanthomonas comprises phytopathogenic bacteria which infect about 400 host species, including a wide variety of economically important plants. Xanthomonas oryzae pv. oryzicola (Fang et al., 1957) Swings et al., 1990 is the causal agent of bacterial leaf streak (BLS) being one of the most destructive bacterial diseases of rice. BLS symptoms are very similar to those of bacterial blight caused by closely related Xanthomonas oryzae pv. oryzae. X. o. pv. oryzae and X. o. pv. oryzicola and often occur in rice f ields simultaneously, so separate leaves may show symptoms of both diseases. The quarantine status and high severity of the pathogen require a highly eff icient, fast and precise diagnostic method. We have developed an assay for Xanthomonas oryzae pv. oryzicola detection using real-time polymerase chain reaction (qPCR) and PCR amplicon sequencing. The DNA samples of X. o. pv. oryzae and X. o. pv. oryzicola were obtained from the collection of CIRM-CFBR (France). To evaluate the analytical sensitivity of the assay, a vector construct based on the pAL2-T plasmid was created through the insertion of X. o. pv. oryzicola target fragment (290 bp). Primers and a probe for qPCR were selected for the hpa1 gene site. They allowed identifying all the strains the sequences of which had been loaded in the GenBank NCBI Nucleotide database before November 11, 2021. The SeqX.o.all sequencing primers were selected for the hrp gene cluster sequence, namely for the nucleotide sequence encoding the Hpa1 protein, the sequencing of which allows for eff icient differentiation of X. oryzae species. The analytical specif icity of the system was tested using the DNAs of 53 closely related and accompanying microorganisms and comprised 100 % with no false-positive or false-negative results registered. The system’s analytical sensitivity was not less than 25 copies per PCR reaction. Its eff icacy has been conf irmed using f ive different qPCR detection systems from different manufacturers, so it can be recommended for diagnostic and screening studies.

## Abstract (ru)

Бактерии рода Xanthomonas Dowson, 1939 поражают около 400 видов растений, в том числе важные сельскохозяйственные культуры. Бактериальная полосатость риса – одно из самых разрушительных заболеваний, вызвано бактериями вида Xanthomonas oryzae pv. oryzicola (Fang et al., 1957) Swings et al., 1990. Сильное сходство симптомов поражения с другим карантинным близкородственным патовариантом – Xanthomonas oryzae pv. oryzae (Ishiyama, 1922) Swings et al., 1990, а также возможность совместного заражения делают визуальную идентификацию невозможной. Карантинный статус и высокая вредоносность патогена требуют высокоэффективного, быстрого и точного метода его диагностики. Целью исследования были разработка и апробация наборов реагентов для выявления бактерии Xanthomonas oryzae pv. oryzicola, вызывающей бактериальную полосатость листьев риса, методом полимеразной цепной реакции в реальном времени (ПЦР- РВ), а также ПЦР с последующим секвенированием ампликонов. В работе изучены образцы ДНК X. o. pv. oryzae и X. o. pv. oryzicola, полученные из коллекции CIRM-CFBR (Франция). Для проверки аналитической чувствитель- ности была создана конструкция на основе вектора pAL2-T с целевой вставкой 290 п. н. Были подобраны и апробированы праймеры и зонд для специфической амплификации фрагмента гена hpa1 методом ПЦР-РВ, позволяющие обнаруживать ДНК X. o. pv. oryzicola. Показана способность с помощью разработанных прай- меров обнаруживать все штаммы X. o. pv. oryzicola, последовательности которых находились в базе данных GenBank NCBI на 11.11.2021. Аналитическая специфичность набора реагентов протестирована на выборке из ДНК, выделенных из 53 близкородственных и сопутствующих организмов, и составила на исследованной выборке 100 %. Ложноположительных и ложноотрицательных результатов не обнаружено. Проверка анали- тической чувствительности показала, что стабильный специфичный сигнал ПЦР-РВ наблюдался при разведе- нии контрольной плазмиды до 25 копий на реакцию. Работоспособность полученного набора реагентов была подтверждена тестированием на пяти приборах для ПЦР-РВ разных производителей, что дает возможность рекомендовать его для проведения диагностических и скрининговых исследований. Праймеры для секвени- рования seqX.o.all были подобраны на последовательность кластера генов hrp, а именно на нуклеотидную последовательность, кодирующую белок Hpa1. Секвенирование выбранного участка позволяет эффективно дифференцировать бактерии вида X. oryzae.

## Introduction

Bacteria Xanthomonas Dowson, 1939 are spread worldwide and able to infect at least 400 kinds of plants including those of high economic importance (Bogdanove et al., 2011; Ryan et al., 2011; Fang et al., 2019). Currently, 27 species of this family have been known, many of which demonstrate high levels of virulence and specificity in certain kinds of plants (Leyns et al., 1984; Ryan et al., 2011; An et al., 2020). Bacterial leaf streak (BLS) is considered to be one of the most devastating diseases caused in rice by Xanthomonas oryzae pv. oryzicola (Fang et al., 1957), Swings et al., 1990 (Soto Suárez et al., 2010).

The disease results in 8 to 32 % of yield loss and is regarded as a serious problem in rice-producing countries (Liu et al., 2014; Jiang et al., 2020). Since the damage done by BLS can seriously threaten the world’s food security (Tang et al., 2000; Lang et al., 2014), Xanthomonas oryzae pv. oryzicola was included in List 1 of harmful quarantine organisms that are not present in the EEU territory as well as in List A1 of the European and Mediterranean Plant Protection Organization (EPPO) that considers the bacteria as quarantine ones( EPPO for the EU under Contract 90/399003. Data Sheets on Quarantine Pests. https://gd.eppo.int/download/doc/530_ds_XANTOR_en [Accessed: 23.11.2021]). Despite the fact that BLS is believed to have been detected for the first time in 1918 in the Philippines, its pathogen was identified only in 1957 in China (Nino-Liu et al., 2006). For the time being, BLS spread is limited to the tropical and subtropical parts of Asia, Northern Australia and a part of Western Africa (EPPO, 2007; Xie et al., 2014; Jiang et al., 2020). The pathogen is absent in the Russian Federation despite cases of infection (Cabi Invasive Species Compendium. Datasheet Xanthomonas oryzae pv. oryzicola (bacterial leaf streak of rice). https://www.cabi.org/isc/ [Accessed: 23.11.2021].) in the southern part of the country and the Russian Far East (EPPO, 2007, 2018). According to the EPPO Reporting Service, no cases of X. o. pv. oryzicola infection have been registered since 1994.(EPPO Global Database. https://gd.eppo.int [Accessed: 23.11.2021].)

Oryza sativa L., 1753, commonly known as Asian rice, is a typical host plant for X. o. pv. oryzicola. In addition, it affects some weed cereals and several other cultivated plants such as Poaceae including Leersia spp., Leptochloa spp., Oryza spp., Paspalum scrobiculatum, Zizania, Zoysia spp. (Ou, 1985; Saddler, Bradbury, 2005; EPPO, 2007). The bacteria mainly spread through infected seeds as well as due to mechanical damage. In case of small plants, infection occurs through wind, raindrops, watering or after contacting infected plant material (Mew et al., 1993).

In plants, X. o. pv. oryzicola reproduce in the substomatal cavity where they get through the stomata to affect the intercellular space of the parenchyma. However, they do not get as far as the xylem and their spread is limited by the mesophyll tissue’s apoplast (Nino-Liu et al., 2006; Triplett et al., 2011; Jacques et al., 2016). The early stage of infection is characterized by small watery interveinal strokes that later transform into bacterial effusion (Mew et al., 1993). The veins act as barriers preventing the pathogen’s further spread and extending a leaf’s affected areas along its length, so they can merge later. In case of severe infection, BLS becomes difficult to differ from the bacterial burn caused by Xanthomonas oryzae pv. oryzae, another quarantine bacterial species. Visual identification can also be complicated by favorable environmental conditions and plant resistance (Swings et al., 1990; Poulin et al., 2014), since both species can infect rice fields at the same time (Mew et al., 1993; Nino-Liu et al., 2006).

The objective of the presented study was to develop and test an assay for genetic detection and diagnostics of the

Xanthomonas oryzae pv. oryzicola pathogen using real time polymerase chain reaction (qPCR) and PCR amplicon sequencing.

## Materials and methods

The presented study was carried out at the Biotechnology Collective Use Center of the All-Russian Research Institute of Agricultural Biotechnology and Syntol LLC. As qPCR positive controls, the DNA samples of the Indian typical strain of X. o. pv. oryzae (2532) and Malaysian pathotype of X. o. pv. oryzicola (2286) from the French Collection of Plant Associated Bacteria (CIRM-CFBP, France) were used. For the last pathotype, a draft whole genome assembly was obtained (Wilkins et al., 2015). X. o. pv. oryzae’s geographic distribution is limited to the territories of Asia, Africa and North America, while that of X. o. pv. oryzicola – to the countries of Asia and Africa. Selecting the target strains, we relied upon the customs statistics of rice import to Russia and according to their data 31.7 % of imported rice in 2018 was supplied by India, followed by Thailand, Pakistan and Kazakhstan.(Agrobusiness Think Tank. https://ab-centre.ru/articles/analiz-importarisa- v-rossiyu-v-2001-2019-gglabelled)

In design of oligonucleotides for qualitative detection of X. o. pv. oryzicola DNA, the hpa1 gene region was used. As many other gram-negative pathogens, X. o. pv. oryzicola has the type III secretion system (T3SS) being a molecular syringe with which the bacteria deliver effector proteins directly into the host cell cytosol (Zhu et al., 2000; Furutani, 2003; Li et al., 2011). The T3SS and its secreted components promote a hypersensitive response (HR) in resistant plants and plants not being the main host for the pathogen. The system is coded as hrp, a hypersensitivity and pathogenicity gene (Cho et al., 2008; Fan et al., 2017), the main operon of which is composed of more than 20 genes in several transcription units that contain the hrp, hrc and hpa genes (Zou et al., 2006; Cho et al., 2008). The oligonucleotides were synthesized by Syntol LLC using their expendables. To design the qPCR and PCR amplicon sequencing reactions, reaction buffer B-009 (Syntol LLC, Russia) was used.

The buffer had the following component concentrations: 3 mmol of MgCl2, 0.25 mmol of dNTP, and 2.5 е. а. of polymerase with antibodies to inhibit ferment activity (Syntol LLC). When designing the oligonucleotides, we made sure the annealing temperature was 60–62 °С for the primers and 64–67 °С – for the probe with 3′-GC-clamp.

The multiparameter analysis of the properties of the selected primers was performed using such online applications as Thermofisher Multiple Primer Analyzer (https:// www.thermofisher.com), Promega Biomath Calculator – Tm for Oligos Calculator (https://worldwide.promega. com), Oligonucleotide Properties Calculator (http://bio tools.nubic.northwestern.edu). The qPCR fluorescence- gglabelled probe incorporated a FAM dye attached to the probe’s 5′ end. The RTQ-1 dye attached to the probe’s 3′ end served as a quencher. The primer concentration in reaction mixture was 800 nM, and 400 nM – in the probe. The qPCR reaction’s repeatability and reproducibility was assessed using the following detection systems: ANK-M (IAI RAS, Russia), QuantStudio 5 (Thermo Fisher Scientific, USA), CFX-96 (Bio-Rad, USA), DTprime 5 (DNA-Technology, Russia), Rotor-Gene 6000 (Qiagen, USA). The obtained results were considered positive if the fluorescence signal level exceeded the threshold of 10 % module difference of the lowest and highest signals.

To verify the analytical sensitivity of the assay, a vector pAL2-T-based structure (Eurogen, Russia) with a 290 bps inclusion of X. oryzae pv. oryzicola was designed. Ligation was carried out after the PCR product was purified using the ColGen DNA purification kit (Syntol LLC). To design the vector-based structure, a T4 DNA ligase buffer (Thermo Fisher Scientific) was used. The plasmid DNA was impregnated into Escherihia coli bacteria (Migula 1895) through thermal shock. The vector’s presence was attested using the PCR-colony method with the standard M13 primers followed by 1.5 % agarose-gel visualization. Plasmid DNA separation was carried out using a PlasGen reagent kit (Syntol LLC). The obtained circular plasmid was processed with the NotI restriction enzyme (ThermoFisher Scientific), its concentration measured in a Quantus fluorometer (Promega Corporation, USA). To test the analytical sensitivity of the designed assay, qPCR to dissolve the plasmid were replicated 2 and 4 times. The analytic specificity of the designed primers and probes was tested using the DNAs of 53 closely related and accompanying microorganisms (Alyapkina et al., 2018).

Bioinformatic analysis and data processing were performed using the UGENE (Unipro, Russia) and AliView (Sweden) software solutions.

To sequence X. o. pv. oryzicola’s DNA, a primer couple including seqX.o.all_F 5′-TCTTTGAACACACAATTC GGCGG-3′ and seqX.o.all_R 5′-TGG AGAATCTCTC CGACGATA-3′ was designed. The amplification program of PCR amplicon sequencing reaction included primary denaturation (5 min at 95 °С); cyclic denaturation (15 s at 95 °С); annihilation (40 s at 60 °С); cyclic elongation (36 cycles of 30 s at 72 °С); final elongation (5 min at 72 °С). The sequencing was carried out using a Nanofor 05 genetic analyzer (IAI RAS).

## Results and discussion

The search for nucleotide sequences in GenBank NCBI found 208 of them to belong to the Xanthomonas family including 20 strains of X. o. pv. oryzicola. During sequence alignment performed in AliView, qPCR oligonucleotides were selected for the regions of the hpa1 target gene conservative to X. o. pv. oryzicola in such a way that the selected primers’ attachment sites were strictly specific and enabled the detection of all the target’s stains the DNA sequences of which had been loaded in the GenBank NCBI database before 11.11.2021.(National Center for Biotechnology Information. http://www.ncbi.nlm. nih.gov [Accessed: 23.11.2021].) Table 1 demonstrates the primer and probe sequencies for X. o. pv. oryzicola diagnostics, selected for its hpa1 gene region

**Table 1.**
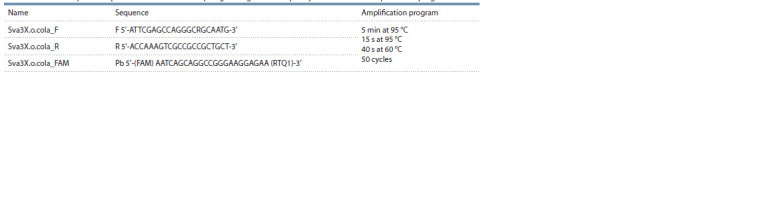
Primer and probe sequencies selected for the hpa1 gene region of X. o. pv. oryzicola and their amplification programs Notе. F – forward primer; R – reverse primer; Pb – probe.

The analytic specificity of the assay was tested using 53 DNA samples of closely related and accompanying microorganisms from different collections that included 9 DNA samples of the bacteria belonging to the Xanthomonas family. The samples were obtained from collections: – of the All-Russian Center of Plant Quarantine and Federal Service for Veterinary and Phytosanitary Surveillance’s divisions: Ralstonia solanacearum 0023, 0027, 0029, 0030, Erwinia amylovora, Clavibacter michiganensis subsp. sepedonicus 0140, 0028, 0244, C. m. subsp. michiganensis 0240, 0241, 0242, 0243, X. o. pv. oryzae 0227, X. phaseoli, Pectobacterium carotovorum subsp. carotovorum 0141, 0168, P. atrosepticum 0142, Dickeya solani, Xylophilus ampelinus 0124, Pantoea stewarti, P. st. subsp. indologenes, P. aglomerance, Candidatus Liberibacter, Acidovorax citrulli; – CIRM-CFBP collection, France: P. st. subsp. indologenes CFBP 3614, C. m. subsp. nebraskensis CFBP 2405, CFBP 3491, Curtobacterium f laccumfaciens pv. f laccumfaciens CFBP 3418, C. f l. pv. poinsettiae CFBP 2403, C. f l. pv. oortii CFBP 1384, X. axonopodis pv. phaseoli CFBP 2534; – Leibniz Institute DSMZ-German Collection of Microorganisms and Cell Cultures GmbH, Germany: X. gardneri DSM 19127, X. perforans DSM 18975, P. wasabiae DSM 18074, X. euvesicatoria DSM 19128, X. vesicatoria DSM 22252, X. translucens pv. translucens DSM 18974, P. cacticida DSM 21821, P. betavasculorum DSM 18076, D. dadantii subsp. dieffenbachiae DSM 18013, D. d. subs. dadantii DSM 18020, D. paradisiaca DSM 18069, D. chrysanthemi DSM 4610, D. zeae DSM 18068, P. c. subsp. odoriferum DSM 22556; – Singerta Company’s collection (Russia): C. m. subsp. michiganensis, Agrobacteria spp., X. campestris pv. campestris, X. translucens pv. translucens; – All-Russian Microorganism Collection of G.K. Skryabin Institute of Microorganism Biochemistry and Physiology (Pushchino, Moscow region, Russia): C. m. subsp. insidiosus ВКМ Ас-1402Т, C. m. subsp. nebraskensis ВКМ Ac-1404Т, Pseudomonas savastanoi ВКМ В-1546; – All-Russian Collection of Industrial Microorganisms of Kurchatov National Research Center – GosNIIgenetika (Moscow, Russia): C. albidum ВКПМ В-1834.

The primers and probe’s analytic specificity for the abovementioned sample set was 100 %. All the samples containing X. o. pv. oryzicola DNA came positive, which was confirmed by sequencing. No false-positive results were registered including those for the DNA of X. o. pv. oryzae, which is a closely related variant of the target pathogen.

**Fig. 1.**
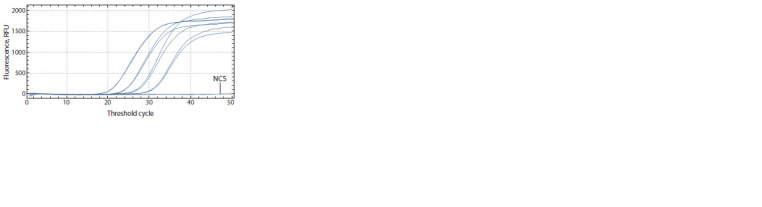
qPCR results, a series of dilutions of a plasmid containing X. o. pv. oryzicola DNA target insertion (0376), FAM detection channel. CFX-96 (Bio-Rad) interface; NCS – negative control sample.

For testing the assay’s analytical sensitivity, the initial concentration of the plasmid with X. o. pv. oryzicola insertion of 13 ng/μl or 3 × 109 copies/μl was used. qPCR in a series of seven dilutions was performed as four 10-time dilutions, first in double repeat, and then in quadruple repeat starting from the fifth series (Fig. 1). After the first dilution, the plasmid concentration reduced to 3 × 105 copies/ μl. Starting from 150 copies, all the following dilution series were additionally titrated as 2 × 10n, 5 × 10n, 7 × 10n in quadruple repeat. For a series of seven 10-time dilutions, the kinetic curve slope comprised A = –2.671, and the correlation ratio, R2 = 0.989. A stable specific signal was observed down to 25 copies in the reaction mixture. In case of 10-time dilution of X. o. pv. oryzicola DNA, the assay showed lower sensitivity – down to 43 copies in the reaction mixture.

**Fig. 2.**
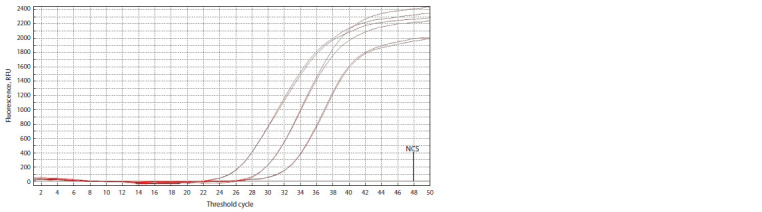
qPCR results, a series of dilutions of a plasmid containing X. o. pv. oryzicola DNA target insertion (0376), FAM detection channel. ANK-M (IAI RAS) interface. NCS – negative control sample.

The designed assay was tested using five qPCR detection systems from Russian and foreign manufacturers (Fig. 2, Table 2). As a matrix, a series of 10-time dilutions of the pathogen’s DNA was applied. The kinetic curve slope comprised А = 3.00–3.67, the correlation ratio, R2 = 0.997– 1.000, and the efficiency, Е = 87–116 %. The threshold value difference comprised ± 1 cycle, which was due to the features of the systems’ design and their threshold cycle computation algorithms.

**Table 2.**
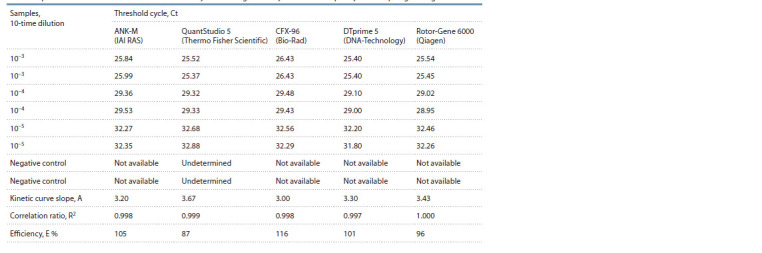
qPCR results obtained in different detection systems using the assay to detect X. o. pv. oryzicola’s hpa1 gene region

To test the primer pair (seqX.o.all_F and seqX.o.all_R) enabling for Sanger sequencing diagnostics, direct PCR was performed. As a matrix, the DNAs of X. o. pv. oryzae (2532) and X. o. pv. oryzicola (2286) were used as well as a 1:1 bacterial DNA mixture to imitate joint infection.

**Fig. 3.**
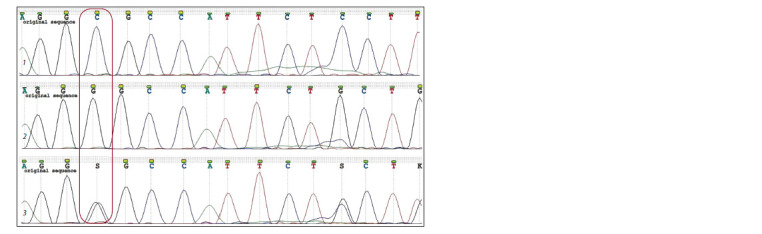
DNA sequence fragments of X. oryzae pv. oryzicola (1), X. oryzae pv. oryzae (2), and a DNA mixture of the two agents (3) that resulted from the sequencing using the seqX.o.all primer pair. The box marks peak matching. Data processed in UGENE v. 38.1 (Unipro).

To differentiate the two closely related bacterial species, a region from 2 288 483–2 288 778 bps characterized by a large number of nucleotide changes relative to the reference sequence CP050113.1 from the NCBI GenBank database was used. Comparison of X. o. pv. oryzae and X. o. pv. oryzicola’s nucleotide sequences and their mixture can be seen in Fig. 3 where peaks C (X. o. pv. oryzicola) and G (X. o. pv. oryzae) match unlike the sequencing results for each of the agents. Bioinformatic analysis of the obtained sequences confirmed they could infect a host both individually and jointly.

Alignment of the obtained nucleotide sequences in the mixture of DNA X .o. pv. oryzae and X. o. pv. oryzicola detected 19 nucleotide changes relative to the reference genome CP050113.1 (Table 3)

**Table 3.**
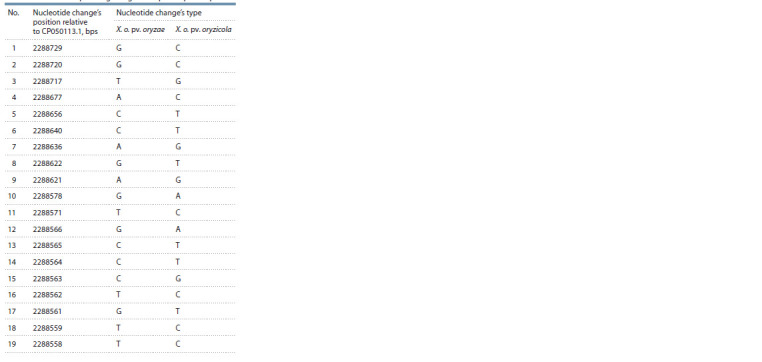
Detected nucleotide changes in the amplified fragment resulted from the sequencing using the seqX.o.all primer pair

**Fig. 4.**
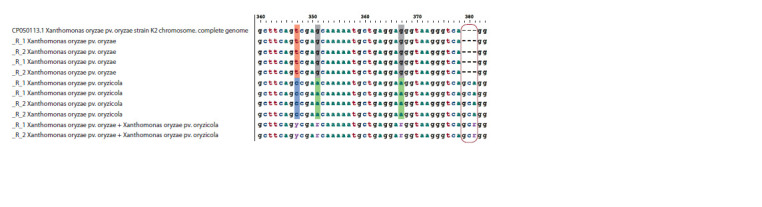
Alignment of the DNA sequencies of X. o. pv. oryzae and X. o. pv. oryzicola and that of their mixture resulted from the sequencing using the seqX.o.all primer pair. The box marks the deletion. The alignments were obtained in AliView v. 1.27 (Sweden).

Apart from the nucleotide changes listed in the Table 3, a three-nucleotide insertion in X. o. pv. oryzicola (in position 2288667 bps) and a three-nucleotide deletion in X. o. pv. oryzicola (in position 2288702 bps) were found relative to the reference X. o. pv. oryzae genome (Fig. 4).

The specific primer placement on X. oryzae enables analyzing pathovariant sequences to detect joint infection by two closely related bacteria and indicate their species.

The obtained nucleotide sequences made it possible to confirm the cultures’ relation to the collected strains in relation to the genome data base. Alignment of the sequences from the data base enabled us to understand certain intraspecific diversity of the X. o. pv. oryzae strains that came from Africa. At the same time, X. o. pv. oryzicola’s diversity was not that high and limited to a single SNP per studied region of the hpa1 gene cluster. Despite the genetic polymorphism of the population of bacterial infections in rice, the designed sequencing and qPCR primers make it possible to detect all isolates irrespectively of the origin of the material included in the Nucleotide NCBI database.

Most of the procedures to detect X. o. pv. oryzae are applicable for X. o. pv. oryzicola as well. Their identification begins with selecting the samples with clear infection symptoms for further cultivation in a nutrient solution. The method has its drawbacks since the colonies of both bacteria grow slowly in isolation media. Another problem is the presence of dominating kinds of bacteria and bacterial antagonists that prevent proper observation of the target ones. Division of X. oryzae’s two pathovariants is possible due to their phenotypical features, induction symptoms, serological test, fingerprinting (polyacrylamide gel electrophoresis) and phagotyping results (Vera Cruz et al., 1984; Benedict et al., 1989; EPPO, 2007).

Restriction fragment length polymorphism changes allow one to observe the almost compete genetic diversity of isolates and their origin (Gonzalez et al., 2007). However, this technique has a number of significant disadvantages such as insufficient sensitivity and specificity; high labor intensity that prevents the method from being used in diagnostic and industrial laboratories. For that reason, PCR has become the key method for detection of the X. oryzae bacteria (Sakthivel et al., 2001).

Current assays allow for X. oryzae detection in general, and further separation into pathovariants requires a standard PCR assay with species-specific primers recommended by the EPPO and All-Russian Center of Plant Quarantine, the results of which are to be sequenced. The EPPO protocol for X. o. pv. oryzae and X. o. pv. oryzicola identification recommends the TXT/TXT-4R primers (Sakthivel et al., 2001; EPPO, 2007; Lang et al., 2010) to be used. To detect X. oryzae DNA using qPCR, it is recommended to use the X.o.F/X.o.R primers devised by the All-Russian Center of Plant Quarantine (Egorova et al., 2014). To separate the two pathovariants, qPCR can be performed using the PF/ PR primers and the TaqMan probe that have been specifically designed to detect X. o. pv. oryzae (Zhao et al., 2007).

In 2021, the All-Russian Center of Plant Quarantine carried out an interlaboratory comparison to detect BLS in rice (21XOO). 16 reference centers and interregional vet laboratories took part and successfully passed a test to detect the disease using the Xanthomonas oryzae pv. oryzicola- RT assay we have designed.

## Conclusion

The designed assay enables the detection of BLS agents in rice. Being a reagent kit for qPCR, is also equipped with the seqX.o.all_F/R primers for PCR amplicon sequencing that detect X. oryzae in cases of individual and joint infection. The system allows for robust screening of quarantinable products and confirms obtained qPCR results with DNA sequencing. The assay has been successfully tested using five qPCR diagnostic systems from different manufactures and can be recommended for diagnostic and screening analysis in research and diagnostic laboratories.

## Conflict of interest

The authors declare no conflict of interest.

## References

Alyapkina Yu.S., Moiseeva M.V., Ksenofontova O.V., Alekseev
Ya.I. Development andvalidation of multiplex real-time
PCR test system for analyzing regulator elements (SsuAra promoter
and E9 terminator) to detect genetically-modified strains
of rape, soybeans, potatoes, and other plants. Izvestiya Timiryazevskoy
Selskokhozyajstvennoy
Akademii = Izvestiya of Timiryazev
Agricultural Academy. 2018;3:5-16. DOI 10.26897/0021-
342X-2018-3-5-16. (in Russian)

An S.Q., Potnis N., Dow M., Vorhölter F.J., He Y.Q., Becker A., Teper
D., Li Y., Wang N., Bleris L., Tang J.L. Mechanistic insights
into host adaptation, virulence and epidemiology of the phytopathogen
Xanthomonas. FEMS Microbiol. Rev. 2020;44(1):
1-32. DOI 10.1093/femsre/fuz024.

Benedict A.A., Alvarez A.M., Berestecky J., Imanaka W., Mizumoto
C.Y., Pollard L.W., Mew T.W., Gonzalez C.F. Pathovarspecific
monoclonal antibodies for Xanthomonas campestris pv.
oryzae and for Xanthomonas campestris pv. oryzicola. Phytopathology.
1989; 79(3):322-328. DOI 10.1094/Phyto-79-322.

Bogdanove A.J., Koebnik R., Lu H., Furutani A., Angiuoli S.V., Patil
P.B., … Brendel V.P., Rabinowicz P.D., Leach J.E., White F.F.,
Salzberg S.L. Two new complete genome sequences offer insight
into host and tissue specificity of plant pathogenic Xanthomonas
spp. J. Bacteriol. 2011;193(19):5450-5464. DOI 10.1128/
JB.05262-11.

Cho H.J., Park Y.J., Noh T.H., Kim Y.T., Kim J.G., Song E.S.,
Lee D.H., Lee B.M. Molecular analysis of the hrp gene cluster
in Xanthomonas oryzae pathovar oryzae KACC10859. Microb.
Pathog.
2008;44(6):473-483. DOI 10.1016/j.micpath.2007.12.
002.

Egorova M.S., Ignatov A.N., Mazurin E.S. Improvement of PCRbased
methods for detecting bacterial blight of rice. Vestnik RosFigsijskogo
Universiteta Druzhby Narodov. Seriya Agronomiya i
Zhivotnovodstvo = RUDN Journal of Agronomy and Animal
Industries.
2014;2:22-27. DOI 10.22363/2312-797X-2014-2-
22-27. (in Russian)

EPPO. Xanthomonas oryzae. Bulletin OEPP/EPPO Bulletin. 2007;
37(3):543-553. DOI 10.1111/j.1365-2338.2007.01162.x.

EPPO. Corrigendum. Bulletin OEPP/EPPO Bulletin. 2018;48(2):
318. DOI 10.1111/epp.12474.

Fan S., Tian F., Li J., Hutchins W., Chen H., Yang F., Yuan X.,
Cui Z., Yang C.H., He C. Identification of phenolic compounds
that suppress the virulence of Xanthomonas oryzae on rice via
the type III secretion system. Mol. Plant. Pathol. 2017;18(4):
555-568. DOI 10.1111/mpp.12415.

Fang Y., Wang H., Liu X., Xin D., Rao Y., Zhu B. Transcriptome
analysis of Xanthomonas oryzae pv. oryzicola exposed to H2O2
reveals horizontal gene transfer contributes to its oxidative stress
response. PLoS One. 2019;14(10):e0218844. DOI 10.1371/
journal.pone.0218844.

Furutani A., Tsuge S., Oku T., Tsuno K., Inoue Y., Ochiai H.,
Kaku H., Kubo Y. Hpa1 secretion via type III secretion system
in Xanthomonas
oryzae pv. oryzae. J. Gen. Plant. Pathol. 2003;
69(4):271-275. DOI 10.1007/s10327-003-0042-2.

Gonzalez C., Szurek B., Manceau C., Mathieu T., Séré Y., Verdier V.
Molecular and pathotypic characterization of new Xanthomonas
oryzae strains from West Africa. Mol. Plant Microbe Interact.
2007;20(5):534-546. DOI 10.1094/MPMI-20-5-0534.

Jacques M.A., Arlat M., Boulanger A., Boureau T., Carrère S., Cesbron
S., Chen N.W., Cociancich S., Darrasse A., Denancé N.,
Fischer-
Le Saux M., Gagnevin L., Koebnik R., Lauber E.,
Noël L.D., Pieretti I., Portier P., Pruvost O., Rieux A., Robène I.,
Royer M., Szurek B., Verdier V., Vernière C. Using ecology,
physiology, and genomics to understand host specificity in
Xanthomonas. Annu. Rev. Phytopathol. 2016;54:163-187. DOI
10.1146/annurev-phyto-080615-100147.

Jiang N., Yan J., Liang Y., Shi Y., He Z., Wu Y., Zeng Q., Liu X.,
Peng J. Resistance genes and their interactions with bacterial
blight/leaf streak pathogens (Xanthomonas oryzae) in rice
(Oryza sativa L.) – an updated review. Rice. 2020;13(1):3. DOI
10.1186/s12284-019-0358-y.

Lang J.M., Hamilton J.P., Diaz M.G.Q., Van Sluys M.A., Burgos
M.R.G., Vera Cruz C.M., Buell C.R., Tisserat N.A.,
Leach J.E. Genomics-based diagnostic marker development
for Xanthomonas oryzae pv. oryzae and X. oryzae pv. oryzicola.
Plant Dis. 2010;94(3):311-319. DOI 10.1094/pdis-94-3-
0311.

Lang J.M., Langlois P., Nguyen M.H., Triplett L.R., Purdie L.,
Holton T.A., Djikeng A., Vera Cruz C.M., Verdier V., Leach J.E.
Sensitive detection of Xanthomonas oryzae pathovars oryzae
and oryzicola by loop-mediated isothermal amplification. Appl.
Environ. Microbiol. 2014;80(15):4519-4530. DOI 10.1128/
AEM.00274-14.

Leyns F., De Cleene M., Swings J.G., De Ley J. The host range
of the genus Xanthomonas. Bot. Rev. 1984;50(3):308-356. DOI
10.1007/bf02862635.

Li Y.R., Zou H.S., Che Y.Z., Cui Y.P., Guo W., Zou L.F., Chatterjee
S., Biddle E.M., Yang C.H., Chen G.Y. A novel regulatory
role of HrpD6 in regulating hrp-hrc-hpa genes in Xanthomonas
oryzae pv. oryzicola. Mol. Plant Microbe Interact. 2011;24(9):
1086-1101. DOI 10.1094/MPMI-09-10-0205.

Liu W., Liu J., Triplett L., Leach J.E., Wang G.L. Novel insights
into rice innate immunity against bacterial and fungal pathogens.
Annu. Rev. Phytopathol. 2014;52:213-241. DOI 10.1146/
annurev-phyto-102313-045926.

Mew T.W., Alvarez A.M., Leach J.E., Swings J. Focus on bacterial
blight of rice. Plant Dis. 1993;77:5-12. DOI 10.1094/PD-
77-0005.

Nino-Liu D.O., Ronald P.C., Bogdanove A.J. Xanthomonas oryzae
pathovars: model pathogens of a model crop. Mol. Plant Pathol.
2006;7(5):303-324. DOI 10.1111/j.1364-3703.2006.00344.x.

Ou S.H. Rice Diseases. Kew: Commonwealth Mycological Institute,
1985;96-101.

Poulin L., Grygiel P., Magne M., Gagnevin L., Rodriguez-R L.M.,
Forero Serna N., Zhao S., El Rafii M., Dao S., Tekete C., Wonni
I., Koita O., Pruvost O., Verdier V., Vernière C., Koebnik R.
New multilocus variable-number tandem-repeat analysis tool
for surveillance and local epidemiology of bacterial leaf blight
and bacterial leaf streak of rice caused by Xanthomonas oryzae.
Appl. Environ. Microbiol. 2014;81(2):688-698. DOI 10.1128/
aem.02768-14.

Ryan R.P., Vorhölter F.J., Potnis N., Jones J.B., Van Sluys M.A.,
Bogdanove A.J., Dow J.M. Pathogenomics of Xanthomonas:
understanding bacterium-plant interactions. Nat. Rev. Microbiol.
2011;9(5):344-355. DOI 10.1038/nrmicro2558.

Saddler G.S., Bradbury J.F. Xanthomonadales ord. nov. In: Brenner
D.J., Krieg N.R., Staley J.T. (Eds.). Bergey’s Manual of
Systematic
Bacteriology. Boston: Springer, 2005;63-122. DOI
10.1007/0-387-28022-7_3.

Sakthivel N., Mortensen C.N., Mathur S.B. Detection of Xanthomonas
oryzae pv. oryzae in artificially inoculated and naturally
infected rice seeds and plants by molecular techniques. Appl.
Microbiol. Biotechnol.
2001;56(3-4):435-441. DOI 10.1007/
s002530100641.

Soto-Suárez M., Gonzalez C., Piégu B., Tohme J., Verdier V. Genomic
comparison between Xanthomonas oryzae pv. oryzae and
Xanthomonas oryzae pv. oryzicola, using suppression-subtractive
hybridization. FEMS Microbiol. Lett. 2010;308(1):16-23.
DOI 10.1111/j.1574-6968.2010.01985.x.

Swings J., Van Den Mooter M., Vauterin L., Hoste B., Gillis M.,
Mew T.W., Kersters K. Reclassification of the causal agents of
bacterial blight (Xanthomonas campestris pv. oryzae) and bacterial
leaf streak (Xanthomonas campestris pv. oryzicola) of
rice as pathovars
of Xanthomonas oryzae (ex Ishiyama 1922)
sp. nov., nom. rev. Int. J. Syst. Bacteriol. 1990;40(3):309-311.
DOI 10.1099/00207713-40-3-309.

Tang D., Wu W., Li W., Lu H., Worland A.J. Mapping of QTLs
conferring
resistance to bacterial leaf streak in rice. Theor. Appl.
Genet. 2000;101:286-291. DOI 10.1007/s001220051481.

Triplett L.R., Hamilton J.P., Buell C.R., Tisserat N.A., Verdier V.,
Zink F., Leach J.E. Genomic analysis of Xanthomonas oryzae
isolates from rice grown in the United States reveals substantial
divergence from known X. oryzae pathovars. Appl. Environ.
Microbiol. 2011;77(12):3930-3937. DOI 10.1128/AEM.
00028-11.

Vera Cruz C.M., Gossele F., Kersters K., Segers P., Van Den
Mooter M., Swings J., De Ley J. Differentiation between Xanthomonas
campetris pv. oryzae, Xanthomonas campestris pv.
oryzicola and the bacterial ‘brown blotch’ pathogen on rice by
numerical analysis of phenotypic features and protein gel electrophoregrams.
J. Gen. Microbiol. 1984;130(11):2983-2999.
DOI 10.1099/00221287-130-11-2983.

Wilkins K.E., Booher N.J., Wang L., Bogdanove A.J. TAL effectors
and activation of predicted host targets distinguish Asian
from African strains of the rice pathogen Xanthomonas oryzae
pv. oryzicola while strict conservation suggests universal importance
of five TAL effectors. Front. Plant Sci. 2015;6:536. DOI
10.3389/fpls.2015.00536.

Xie X., Chen Z., Cao J., Guan H., Lin D., Li C., Lan T., Duan Y.,
Mao D., Wu W. Toward the positional cloning of qBlsr5a, a QTL
underlying resistance to bacterial leaf streak, using overlapping
sub-CSSLs in rice. PLoS One. 2014;9(4):e95751. DOI 10.1371/
journal.pone.0095751.

Zhao W.J., Zhu S.F., Liao X.L., Chen H.Y., Tan T.W. Detection of
Xanthomonas oryzae pv. oryzae in seeds using a specific TaqMan
probe. Mol. Biotechnol. 2007;35(2):119-127. DOI 10.1007/
BF02686106.

Zhu W., MaGbanua M.M., White F.F. Identification of two novel
hrp-associated genes in the hrp gene cluster of Xanthomonas
oryzae pv. oryzae. J. Bacteriol. 2000;182(7):1844-1853. DOI
10.1128/JB.182.7.1844-1853.2000.

Zou L.F., Wang X.P., Xiang Y., Zhang B., Li Y.R., Xiao Y.L.,
Wang J.S., Walmsley A.R., Chen G.Y. Elucidation of the hrp
clusters of Xanthomonas oryzae pv. oryzicola that control the
hypersensitive response in nonhost tobacco and pathogenicity
in susceptible host rice. Appl. Environ. Microbiol. 2006;72(9):
6212-6224. DOI 10.1128/AEM.00511-06.

